# From Muscles to Wires: Report of Two Cases and Literature Review on COVID-19 Vaccination and Cardiac Conduction Disturbance

**DOI:** 10.7759/cureus.18805

**Published:** 2021-10-15

**Authors:** Mohamed Elhassan, Hasan Ahmad, Mohamed Mohamed, Ola Saidahmed, Ahmed E Elhassan

**Affiliations:** 1 Cardiology, Royal Derby Hospital, Derby, GBR; 2 Cardiology, Countess of Chester Hospital, Chester, GBR; 3 Gastroenterology, Royal Victoria Infirmary, Newcastle upon Tyne Hospitals NHS Foundation Trust, Newcastle upon Tyne, GBR; 4 Internal Medicine, Royal Derby Hospital, Derby, GBR; 5 Internal Medicine, South Tees Hospitals NHS Foundation Trust, Middlesbrough, GBR

**Keywords:** covid-19, permanent pacemaker implantation (ppm), covid-19 vaccine, myocarditis, cardiac conductive disturbance

## Abstract

Since the end of 2020, several vaccines have become available as part of the global efforts to contain the adverse health outcomes of the coronavirus disease 2019 (COVID-19) pandemic. Although research has confirmed their safety on large scales, several post-marketing reports have revealed some rare cardiovascular side effects. Towards the end of the first half of 2021, multiple reports indicate possible links between COVID-19 vaccines (both mRNA-based vaccine and vector-based vaccines) and myopericarditis. Nevertheless, cardiac conduction disease in this context has only rarely been reported.

In this report, we present two cases of probable vaccination-induced cardiac conduction disturbances along with a thorough literature review. In addition, we discuss probable pathophysiological mechanisms and insights into the suggested areas for future research. To our knowledge, these are the first published cases to result in permanent pacemaker implantation.

## Introduction

In March 2020, the World Health Organization (WHO) announced coronavirus disease 2019 (COVID-19) the second pandemic of the 21st century [1]. Since its emergence in Wuhan, the capital of Hubei province in China, and as of August 2021, the pandemic has claimed the lives of over four million people, as registered by the WHO statistics to the date of writing this report [2]. COVID-19 has had a significant impact, both directly and indirectly, on other aspects including socioeconomic and policy implications globally [3]. Since then, research has been ongoing not only on aspects relating to the pathophysiology, virulence, and mechanisms of viral infection but also for developing effective therapies and vaccines [4].

Since the end of 2020 and the beginning of 2021, several vaccines have been found promising in terms of efficacy and safety [5-7]. However, a few potential cardiovascular side effects have been reported (e.g., myocardial infarction, deep vein thrombosis, and pulmonary embolism) [8]. More recently, there have been increasing reports suggesting a possible role of COVID-19 vaccines in the development of myocarditis, pericarditis, and myopericarditis as complications with variable outcomes [9-11].

Although numerous reports have linked COVID-19 vaccination to myopericarditis [9-11], to date, cardiac conduction disturbance (CCD) (whether isolated or as part of myocarditis) has only rarely been reported. In this report, we thoroughly present and discuss the cases of two patients presenting with some forms of CCD as probable complications to COVID-19 vaccines. A thorough systematic literature search is also included in this paper. This involved Embase and PubMed databases. An advanced search was conducted in both databases using relevant keywords, MeSH terms, and Emtree. To our knowledge, these are the first published cases to end up requiring permanent pacing.

## Case presentation

Case One

**Figure 1:**
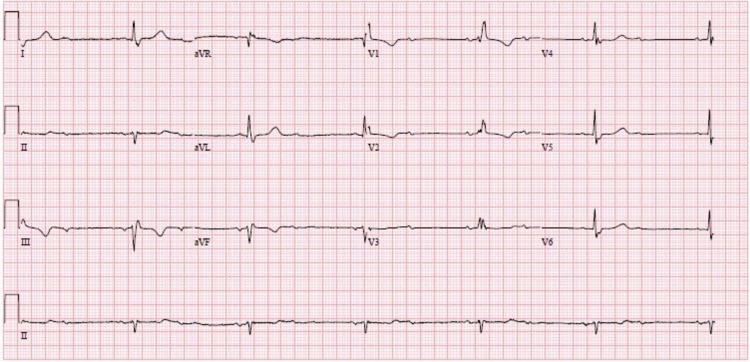
A 12-lead ECG showing evidence of LAFB, RBBB, and 2:1 AV conduction block. ECG: electrocardiogram; LAFB: left anterior fascicular hemiblock; RBBB: right bundle branch block; AV: atrioventricular

An 89-year-old fit and independently mobile Caucasian female was admitted to the Coronary Care Unit (CCU) one day after she had received her first COVID-19 AstraZeneca (ChAdOx1 nCoV-19) vaccine. She had a remote history of total knee replacement but otherwise did not have a significant previous cardiac history (apart from previous electrocardiograms [ECGs] showing a bifascicular block with no symptoms). The sequence of events started a few hours (estimated to be three to four hours) after taking the vaccine. She started to experience headaches, nausea, non-specific body aches, fever, and chills. She was not sure if she had chest pain but reported probable constricting chest discomfort as part of the generalized body aches. A few hours after the resolution, these symptoms were followed by expressive dysphasia and pre-syncopal symptoms, for which she was rushed to the emergency department as a possible stroke presentation. Upon arrival, all her symptoms had completely resolved. A computed tomography (CT) scan of the brain was unremarkable. However, she was noted to be bradycardic with a heart rate of approximately 30 beats per minute. The rest of her observations included a blood pressure of 182/79 mmHg, oxygen saturation of 96% on room air, the highest recorded temperature of 37.8°C, and a respiratory rate of 16 breaths per minute. Cardiovascular and respiratory examinations were unremarkable. Initial laboratory investigations were within normal parameters. These included full blood counts, renal function tests and electrolytes, C-reactive protein, coagulation profile, liver function tests, thyroid function tests, HbA1c, calcium, magnesium, phosphate, and blood gas analysis. Serum troponin-T was mildly elevated at 14 ng/L (using high-sensitivity assay). This was the only sample taken and there were no serial measurements of this test as this was deemed to be below the threshold of significance for acute coronary syndrome as per the hospital protocol. An initial ECG (Figure 1) showed evidence of left anterior fascicular hemiblock (LAFB) and right bundle branch block (RBBB) with two to one (2:1) atrioventricular (AV) conduction block in the form of second-degree AV conduction block, confirming a form of a trifascicular block. It was noted that the patient had old ECGs showing LAFB and RBBB only (a bifascicular block).

The patient was monitored closely in the CCU overnight. A transthoracic echocardiogram (TTE) performed the next day showed normal ejection fraction (>55%) with only grade one diastolic dysfunction which was insignificant and was attributed to the patient’s age. Otherwise, there was neither evidence of regional wall motion abnormalities nor any significant interventricular dys-synchrony.

As the patient remained in the above-mentioned rhythm (2:1 AV conduction block) overnight with some episodes of probable complete heart block (CHB) on telemetry, a decision was taken the next day to proceed with implanting a dual-chamber permanent pacemaker (PPM).

It is worth mentioning that the patient was not on any negative chronotropic agents and did not have any obvious modifiable risk factors or causes for bradyarrhythmia. Cardiac magnetic resonance imaging (MRI) was neither feasible nor safe at the time (due to the acuity of the presentation). Moreover, in our view, it would not have had any impact on the management plan.

The patient was discharged home the same day after PPM insertion. She continued to have pacing follow-up appointments. The first one was two months after discharge. At the two-month follow-up, a well-functioning pacemaker was observed. Further investigation showed an underlying rhythm of intermittent 2:1 AV block. The atrial pacing percentage was 1.7% while the ventricular pacing was 37%. Another pacing investigation after four months showed an underlying sinus rhythm with non-pacing-dependent ventricular activity. The patient also underwent a carotid Doppler at this time as an outpatient study, which did not show significant lesions.

Case two

An 85-year-old Caucasian male was known to have depression, anxiety, glaucoma, and a history of right forehead basal cell carcinoma excision six years ago. He had good functional baseline and normal mobility. His regular medications included mirtazapine 15 mg once a day, Timolol eye drops, and atorvastatin 20 mg once a day. He was allergic to penicillin with an undisclosed nature.

He presented to our emergency department with gradually worsening breathlessness, effort intolerance, and episodes of dizziness at rest over a course of 10 days after taking his first dose of the COVID-19 Pfizer-BioNTech (BNT162b2) vaccine. On direct questioning, the patient denied having had any fever, chills, or body aches immediately after the vaccination.

His initial observations upon arrival were a regular heart rate of 36 beats per minute, a temperature of 36°C, a respiratory rate of 17 breaths per minute, and a blood pressure of 180/82 mmHg. He had normal cardiovascular and chest examinations. He did not have lower limb edema, and his jugular venous pressure was not raised. Laboratory investigations included full blood counts, renal function and electrolytes, C-reactive protein, cardiac enzymes, coagulation profile, liver function tests, thyroid function tests, HbA1c, calcium, magnesium, phosphate, and blood gas analysis, which were largely unremarkable.

**Figure 2:**
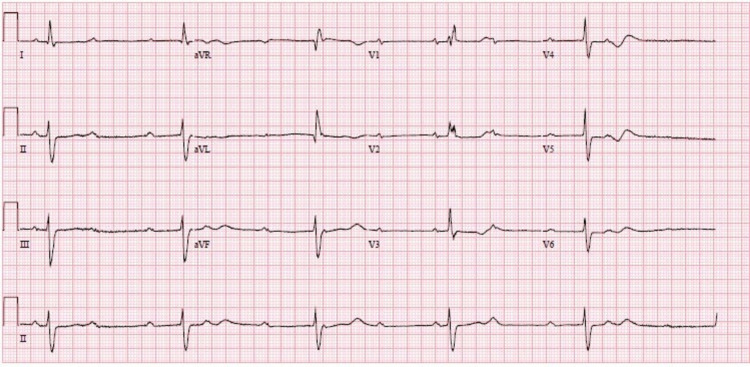
A 12-lead ECG showing evidence of CHB. ECG: electrocardiogram; CHB: complete heart block

A chest X-ray did not show any obvious abnormalities. He underwent a 12-lead ECG (Figure 2) that showed evidence of CHB with a QRS duration of 136 milliseconds showing RBBB morphology. There was also evidence of LAFB. The patient also underwent a TTE which showed normal left ventricular function (ejection fraction > 55%).

Eventually, the patient was admitted to the CCU with telemetry monitoring and had a dual-chamber PPM implanted the next morning. He had an uneventful recovery and was discharged home the same day after satisfactory pacing checks.

On this occasion, the patient did not have any old ECGs to compare and identify if there were any baseline conduction abnormalities. Follow-up pacing investigation after two months showed a consistent baseline rhythm of CHB with a ventricular pacing percentage of almost 99% (pacing-dependent). Another follow-up at four months from the date of the PPM insertion confirmed the same findings.

## Discussion

The cardiac conduction system of the heart is a specialized system that primes organized and coordinated cardiac muscle contraction resulting in effective systole and diastole. This system consists of the sinoatrial SA node, the AV node, the bundle of His, and the ventricular conduction system (including right, left bundle branches, and Purkinje fibers) [12]. Cardiac conduction disease/disturbance is a group of variable etiologies. This can be broadly categorized into structural or functional pathologies, each of which can be further subdivided into acquired or congenital causes. Examples of the above include Lev’s disease (relating to aging), electrolyte abnormalities, certain drug causes, ischemic pathologies, inflammatory (myocarditis, autoimmune, or infectious), and some inherited or congenital channelopathies [13].

Myocarditis, one of the acquired causes of CCD, is defined as cellular inflammatory infiltration with or without necrosis of the myocytes [14]. The European Society of Cardiology differentiates between myocarditis and inflammatory cardiomyopathy. They characterize inflammatory cardiomyopathy as a histological and functional diagnosis comprising myocarditis in association with clinical evidence of cardiac dysfunction [15]. Due to the variable clinical presentations ranging from subclinical to more overt severe presentations resulting in significant mortality and morbidity, myocarditis diagnosis can sometimes be challenging [10]. The pathogenesis involves variable mechanisms, which include direct cardiac injury (e.g., cardiotropic viruses such as Adenoviruses and Enteroviruses), indirect injury via an immune-mediated response (e.g., HIV, Influenza A and B viruses, and hepatitis C virus), or both (e.g., Coronaviridae family including the Middle-East respiratory syndrome coronavirus, severe acute respiratory syndrome coronavirus (SARS-CoV) and SARS-CoV-2) via direct invasion and angiotensin-converting enzyme 2 tropism [10].

Lazaros et al. postulated a cytokine-mediated immune response as the culprit for COVID-19 vaccine-induced myocarditis [10]. Although the authors have limited this to the reactogenicity of the mRNA vaccines only, there is growing evidence suggesting possible links between myocarditis and vector-based vaccines (particularly the COVID-19 AstraZeneca (ChAdOx1 nCoV-19) vaccine). These are reports by the United Kingdom Medicines and Healthcare Products Regulatory Agency as part of the open “Yellow card” reporting system [16,17]. It remains unclear whether a similar mechanism exists for myocarditis related to vector-based vaccines. Indeed, our first case (Case one) was vaccinated using a vector-based vaccine.

As mentioned above, our first patient presented with prodromal symptoms shortly after vaccination suggesting a probable “cytokine storm.” Although it was unfortunate that an MRI scan was not feasible, the presence of chest discomfort and mild troponin leak (above the 99th centile of the reference range) might arguably point towards a myocarditic process. In the literature review, we identified a few cases of CCD in the context of vaccination-induced myocarditis. In the first one, among several cases, Marshall et al. reported the case of a 16-year-old male who developed fever, fatigue, and loss of appetite two days after his second dose of the COVID-19 Pfizer-BioNTech (BNT162b2) vaccine. The authors reported evidence of junctional escape rhythm with AV dissociation. There was also evidence of ST-segment elevation. The patient eventually recovered completely [18]. The second case was reported by Muthukumar et al. They reported the case of a 52-year-old male who developed fever, myalgias, and headache after the second dose of mRNA-1273 (Moderna) COVID-19 vaccine. After the third day, he developed substernal chest pain. An ECG showed evidence of incomplete RBBB, left axis deviation (LAD), and no evidence of ST-segment or T-wave changes. The patient was reported to have recovered with no sequelae [19]. The third case was presented by Tano et al. as part of a case series [20]. They reported the case of a 15-year-old male who presented with intermittent chest pain three days after receiving the second dose of the COVID-19 Pfizer-BioNTech (BNT162b2) vaccine. The ECG did not show non-specific intraventricular conduction delay with slight ST-segment depression that was transient. Similar to the two reported cases by Marshall et al. and Muthukumar et al., our first patient had a fever and showed prodromal symptoms before the onset of myocarditis. The main differences noted, however are: first, our Case one patient developed these symptoms after the COVID-19 AstraZeneca (ChAdOx1 nCoV-19) vaccine. Second, the complex of symptoms occurred after the first dose of vaccination and not the second dose. On the other hand, similar to the patient reported by Tano et al., our Case two patient did not have a fever.

It is interesting to note that although both of our patients ended up needing PPM insertion, subsequent pacing follow-up revealed almost complete pacing dependence only in our second patient. Therefore, It is unclear whether this represents the potential reversibility of the inflammatory process with the recovery of the conductive system function or the co-existence of a progressive CCD (e.g., Lev’s disease) particularly in the second case. Hence, the question of whether vaccination-induced CCD is a matter of “triggering” versus “confounding factor” remains to be answered. Certainly, our first patient had previous ECGs confirming background CCD.

In the context of the current pandemic, we realistically acknowledge that the benefits of the COVID-19 vaccination outweigh the risks reported so far. On a clinical level, however, we suggest taking into consideration individual patient factors that might prove significant in the risk-benefit clinical reasoning (e.g., baseline and old ECGs showing CCD, previous similar reactions, and history of myocarditis). These should not be viewed as contraindications to vaccination but rather should raise awareness about such potential side effects.

## Conclusions

As per our case presentation and discussion, we believe that CCD holds the potential of being a rare complication of COVID-19 vaccines, especially in the context of worldwide vaccination inertia. It is yet unclear what underlying mechanisms play a role (whether immune-mediated or otherwise). We acknowledge that COVID-19 vaccination programs remain vital to hinder the progression of the pandemic. However, physicians should have a high degree of vigilance and awareness of potential side effects. In the context of myocarditis and CCD, it might be worthwhile paying attention to some factors such as baseline cardiac conduction and old ECGs. Further research is required to clarify this further.
